# Preparation, Characterization, and In Vitro Stability Analysis of Deer Sinew Peptide-Zinc Chelate

**DOI:** 10.3390/foods14122131

**Published:** 2025-06-18

**Authors:** Shan Yang, Tianyuan Liu, Weijia Chen, Ying Zong, Jianan Geng, Yan Zhao, Zhongmei He, Rui Du

**Affiliations:** 1 College of Traditional Chinese Medicines, Jilin Agricultural University, No. 2888, Xincheng Street, Changchun 130118, China; 15761404093@163.com (S.Y.); 18186812352@163.com (T.L.); chenweijia_jlau@163.com (W.C.); zongying7699@126.com (Y.Z.); gengjianan@jlau.edu.cn (J.G.); zhaoyan@jlau.edu.cn (Y.Z.); 2 College of Pharmacy, Yanbian University, 977 Park Road, Yanji 133002, China

**Keywords:** deer tendon protein, peptides-zinc chelate, response surface methodology, structural characterization, simulated gastrointestinal digestion

## Abstract

Novel peptide-zinc chelates (DSPs-Zn) with a zinc content of 186.94 mg/g were synthesized from deer tendon peptides at pH 6, 60 °C, 60 min, and peptide-zinc mass ratio of 1:3. Ultraviolet-visible absorption spectroscopy (UV) and Fourier transform infrared spectroscopy (FTIR) demonstrated that the chelation sites of the deer tendon polypeptides (DSPs) with zinc ions were located at the carboxyl oxygen and amino nitrogen atoms of the peptides. Amino acid analysis showed that aspartic acid, glutamic acid, lysine, and arginine play important roles in the chelation process. In vitro simulated gastrointestinal digestion studies showed that DSPs-zinc exhibited higher stability than zinc sulfate and zinc gluconate in the pH range 2–8 and in a simulated gastrointestinal digestion environment. The above experimental results suggest that DSPs-Zn has the potential to be used as a novel zinc nutritional supplement.

## 1. Introduction

Zinc has a variety of biological functions and is one of the body’s essential trace elements. It is used as a catalytic cofactor in more than 300 different metal catalysts and plays a key role in the synthesis and breakdown of many chemical substances [1]. Zinc additionally plays an impact in the enzyme activity of polymerases in DNA replication and transcription [2]. In addition, zinc requires exposure to preserve every aspect of the body’s development and growth, the immune system, and brain function [3]. Other clinical signs of zinc deficiency include development retardation, immune dysfunction, DNA damage, and oxidative damage that results in chronic inflammation [4]. According to statistics, approximately 2 billion individuals globally experience various levels of zinc insufficiency, and about 17% of the population is mainly associated with the consumption of foods with insufficient zinc content [5]. It is pertinent to highlight that the large proportion of developing countries stems from the fact that low-income populations in developing countries cannot afford foods containing absorbable varieties of zinc [6] and are more likely to consume plant foods, which interfere with zinc absorption due to the presence of phytic acid, which has an excellent complexing ability for most metal ions, and which complexes with zinc ions in the digestive tract to form insoluble complexes [7]. Therefore, the improvement of zinc deficiency through supplementation or fortification of foods has been mainstreamed into research. The initial use of inorganic zinc salts was eliminated because of their poor solubility and their impact on the taste of food. Organic acid zinc supplements, on the other hand, can contribute to interactions between nutrients, leading to a decrease in the bioavailability of some nutrients such as zinc [8]. Furthermore, peptides have become the main emphasis of public attention. Food-derived peptide structures containing metal ligands such as His, Cys, Asp, Glu, and Ser (as well as phosphorylated Ser) can act as dietary zinc carriers [9], binding to zinc to form soluble zinc complexes and preventing phytates from complexing with zinc [10], hence enhancing zinc’s bioavailability and digesting stability in the gastrointestinal tract [11]. In addition, peptide zinc chelates are absorbed intact in the intestinal tract in a soluble form using a special transport channel, which greatly facilitates the efficient absorption of zinc [12]. Li et al. [13] found that oyster-derived zinc-binding peptides can promote intestinal absorption of zinc through zinc chelation modification via the Plastein reaction. Whey-derived peptide-chelated zinc improves in vitro gastric stability and zinc bioavailability [14]. A study conducted by Suo et al. [15] demonstrated that broilers fed both regular corn-soybean meal feed and zinc-methionine chelate feed had significantly higher relative bioavailability of zinc from the chelate form compared to those fed exclusively on the regular corn-soybean meal diet. This has important implications for the search of effective and side effect-free ingredients as alternatives for the prevention of zinc deficiency and at the same time as novel nutritional supplements to improve the bioavailability of zinc in the gastrointestinal tract.

The large surplus of deer tendon (DS) compared to other deer product resources is mainly due to its low demand and utilization as a by-product of deer processing. Developments in the field of food and dietary supplements have led to the full recycling of the resource, and deer tendon has attracted attention due to its richness in collagen (82.12%) and inorganic elements [16]. Additionally, deer tendon peptides have the ability to absorb calcium [17] along with anti-osteoporotic properties [18]. Based on the previous work, deer tendon peptides chelate with calcium to promote calcium absorption, which potentially suggests that deer tendon peptides have good metal chelating activity, but whether they have the potential to chelate zinc ions and promote zinc absorption is currently unknown.

Consequently, the current study was conducted in order to determine the optimal preparation process for zinc chelates of deer tendon peptides by response surface methodology. The structural and compositional properties before and after chelation were investigated using modern instrumental analytical techniques to assess the chelates’ physicochemical characteristics and chelating ability in addition to the stability and bioavailability of the zinc peptide chelates in many settings.

The results of the study may provide a scientific basis for the development of new zinc nutritional supplements and promote the development and utilization of deer tendon peptide resources.

## 2. Materials and Methods

### 2.1. Materials and Chemicals

Deer tendon (Jilin Agricultural University, Changchun, China). Genye Biotechnology Co. supplied the alkaline protease (200 μ/mg, Shanghai, China). The remaining compounds utilized in this investigation were analytical grade.

### 2.2. Preparation of Deer Tendon Peptides

Peptides from deer tendon were made with certain adjustments in accordance with Cao et al. [19]. Deer tendon was soaked with citric acid at a concentration of 7% in a solid–liquid proportion of 1:25, and the solution was subsequently heated to 100 °C to dissolve it, filtered through skimmed gauze to remove residual impurities, and centrifuged at 9000 rpm for 20 min at 4 °C to take the supernatant. Alkaline protease was used to extract the DSPs, and the subsequent conditions for hydrolysis were established: enzyme adding 1000 U/g, hydrolysis reaction time of 5 h, temperature of 53 °C, and pH of 9.4. The supernatant should be collected after 15 min of heating at 100 degrees, and then freeze-dried as DSPs.

### 2.3. Optimization of Zinc Chelation Conditions for Deer Tendon Peptides

The effects of four factors, namely, peptide-zinc mass ratio, pH, temperature and time, on the zinc content in DSPs-Zn were investigated by using a one-way test method under the condition of controlling a single variable with zinc content as the detection index. The lyophilized DSPs were dissolved in ultrapure water. They were mixed with Zn_2_SO_4_ according to varying mass proportions (1:0.5–1:4). Subsequently, pH was adjusted and modified to an assortment of 4 to 8, and the reaction was conducted at different temperatures (40–80 °C) (40–80 min). Anhydrous ethanol equivalent to five times that of the reactants was introduced to facilitate the precipitation of DSPs-Zn. Following centrifugation at 4000 revolutions per minute for a duration of 20 min, the resulting precipitates were harvested, subjected to lyophilization, and subsequently designated as DSPs-Zn chelates. The preparation conditions for DSPs-Zn were optimized through a one-factor experimental approach utilizing the Box-Behnken design methodology. The preceding one-way experiment demonstrated that time did not influence the chelating capacity of DSPs. Consequently, response surface experiments were created to investigate how the zinc concentration (Y, mg/g) in the chelate is affected by three independent variables: pH (C), peptide-zinc mass ratio (B), and chelation temperature (A). Multiple regression analysis of the data obtained from experimental data is displayed in Table 1 using Design-Expert.

### 2.4. Analysis of Zinc Content

For the determination of zinc concentrations, a slight modification of the strategy described by Sun et al. [20] has been employed to determine the zinc concentration. DSPs-Zn was first dissolved in 50 milliliters of ultrapure water. Then, 5 mL of an ammonia-ammonium chloride buffer (pH 10) (Shanghai McLean Biochemical Technology Co., Ltd.,Shanghai, China) and two to three drops of chromium black T (Shanghai Yuanye Biotechnology Co., Ltd., Shanghai, China) were added to a 10 mL aliquot of this solution. After that, 0.01 mol/L EDTA (Shanghai Yi Miao Chemical Technology Co., Ltd., Shanghai, China) was added to the resultant solution until a purple to blue color shift was seen. The zinc content of DSPs-Zn was then calculated using the formula below:

X = (M × C × V × 5)/m × 100%

In this context, X represents the zinc concentration in DSPs-Zn, M is the fraction of zinc, V is the amount of volume of the EDTA solution used, and m is the total quantity of DSPs-Zn.

### 2.5. Characterization of DSPs Versus DSPs-Zn Chelates

#### 2.5.1. UV-Vis Analysis

Deionized water was used to dissolve DSPs and their derivative DSPs-Zn, creating a sample solution containing a dosage of 1 mg/mL. A calibration procedure was performed on a UV-visible spectrophotometer (UV-1900i, Shimadzu, Kyoto, Japan) using deionized water as the control sample. Continuous scanning analysis was carried out between 200 and 400 nm in wavelength.

#### 2.5.2. FT-IR Analysis

DSPs in addition to DSPs-Zn (2 mg each) were thoroughly combined with a suitable quantity of KBr, pressed into discs using a mold, and the structures of the DSPs and DSPs-Zn chelates were characterized using an infrared spectrometer (Ll, PerkinElmer, London, UK) within the 400–4000 cm^−1^ wavelength region.

#### 2.5.3. Analysis of Particle Size and Zeta Potential

We created DSP and DSPs-Zn solutions with a 1 mg/mL concentration. Particle size and zeta potential have been assessed with the nanoparticle size potentiometer (Zetasizer Nano ZS Zen 3600, Malvern PANalytical Instruments Ltd., Nottingham, UK).

#### 2.5.4. SEM and EDS Analysis

Vacuum analysis of the DSPs and DSP-Zn nanoparticles has been conducted employing a scanning electron microscope (SEM, APreo, Thermo Fisher Scientific Co. Ltd., Waltham, MA, USA). There was calibration of the accelerating voltage, and the sample height was appropriately adjusted, the sample was positioned, the magnification was adjusted and focused, and the scan was scanned to obtain microstructural maps and analyze their surface elemental compositions by energy dispersive spectrometer (EDS, Oxford Instruments Ltd., Oxfordshire, UK) to examine the surface’s elemental makeup.

#### 2.5.5. Molecular Weight Distribution

Using gel permeation chromatography, the molecular weight distribution was ascertained by dissolving the sample to be examined in a mobile phase made with 0.1 mol/L NaNO_3_ solution. A PL aquagel-OH MIXED-H column (dimensions: 8 μm, 300 mm × 7.5 mm) at a detection wavelength of 220 nm and a flow rate of 1 mL/min.

#### 2.5.6. Amino Acid Composition

Samples designated for testing were hydrolyzed using 6 mol/L hydrochloric acid at 110 °C for 24 h and then analyzed for amino acid composition using an amino acid analyzer (LA8080, Hitachi, Tokyo, Japan).

### 2.6. Solubility Analysis of Zinc at Different pH Conditions

Zinc sulfate and DSPs-Zn solubility were evaluated using a modified version of the methods outlined in Xu et al. [21]. Zinc sulfate and DSPs-Zn were dissolved in deionized water with varying pH levels (2–8). Following 10 min of incubation at 37 °C and 10 min of centrifugation at 4000× *g*, the content of zinc in the supernatant was measured using an atomic absorption spectrophotometer.

### 2.7. Simulated Gastrointestinal Digestion Experiment

The technique outlined by Sun et al. [22] was used to replicate the gastrointestinal digestion process. Specifically, Pepsin (40 mg) was dissolved in hydrochloric acid solution to simulate gastric juice. Additionally, porcine bile salts (3 g), trypsin (0.45 g), and sodium hydroxide (6.25 g) were submerged in deionized water to mimic intestinal fluids. Zinc gluconate, zinc sulphate, and DSPs-zinc were left to react for 30 min at 37 °C. During the gastric digestion phase, aliquots were collected at 30 min intervals over a period of 90 min. Following the gastric digestion, add the enteric solution after adjusting the pH to 6.8. Similarly, during the enteric digestion phase, aliquots were taken at 30 min intervals for a duration of 150 min. All aliquots collected throughout the procedure were subjected to heating at 100 °C for 20 min to halt enzymatic activity. Aliquots were centrifuged for 20 min at 6000× *g*, and the supernatant was gathered so that an atomic absorption spectrophotometer could determine the zinc level. The formula for calculating zinc solubility is as follows:

*Zinc solubility (%) = (zinc in supernatant (mg)/total zinc in solution (mg)) × 100*

### 2.8. Statistical Analysis

The above data were statistically analyzed using IBM SPSS software for Windows version 24.0 (IBM Corp., Armonk, NY, USA) and are expressed as mean ± SD. Data were compared between groups by one-way analysis of variance (ANOVA) and Tukey’s test. *p*-values < 0.05 were considered to indicate statistically significant differences. In addition, Origin 2025 was selected for graphical visualization.

## 3. Results and Discussion

### 3.1. Process Optimization of DSPs-Zn Preparation Conditions

#### 3.1.1. One-Factor Analysis

**Figure 1:**
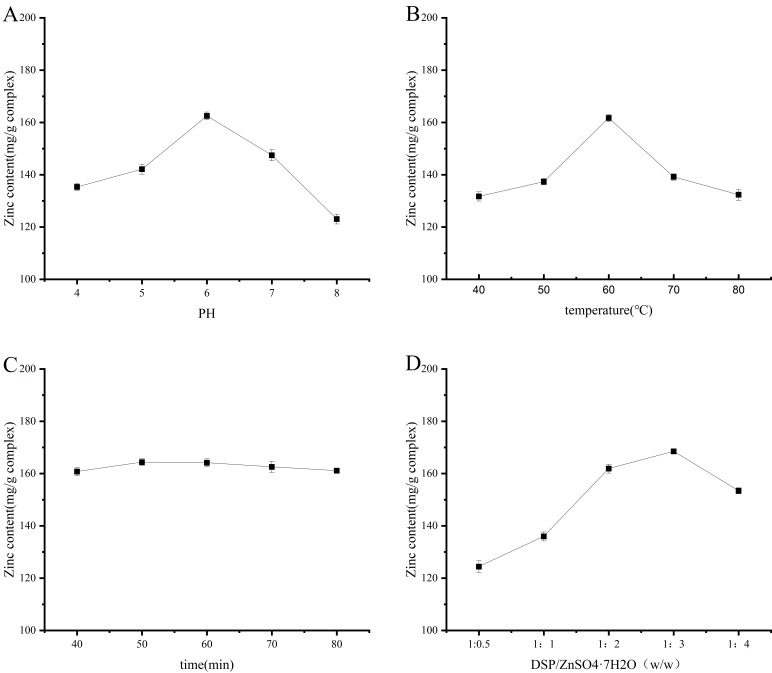
Graphs illustrate the influence of pH (**A**), temperature (**B**), duration (**C**), and the mass ratio of DSPs to ZnSO_4_-7H_2_O (**D**) on the zinc binding capacity. Note: DSPs: deer tendon polypeptides; DSPs-Zn: deer tendon polypeptide zinc chelates.

Figure 1 illustrates the zinc concentration in DSPs-Zn under varying conditions. The data indicate a substantial increase in zinc content as the pH rises from 4 to 6, peaking at pH 6. One possible explanation is that at lower pH, H^+^ in solution dissociates from the -NH_2_ and -COOH functional groups [23], facilitating the interaction of the electron-donating groups on the DSPs with the metal ions and favoring the formation of chelates [24]. In cases where the pH exceeded 6, the high concentration of OH- would first form a white precipitate of Zn(OH)_2_ with zinc ions, which resulted in a notable reduction in DSPs’ ability to bind to zinc ions [25]. Zinc content of DSPs-Zn increased significantly as the temperature increased from 40 °C to 60 °C, as illustrated in Figure 1B. The reactivity of peptides with metals is a heat-absorbing reaction, and a proper increase in temperature facilitates the movement of molecules and provides more energy to maintain and enhance the stability and efficiency of the DSPs-Zn synthesis. A notable decrease in the zinc concentration of DSPs-Zn was noted at temperatures above 60 °C, which could be related to the partial denaturation of DSPs brought on by high temperatures as well as the breakdown of DSPs-Zn chelates [26]. The concentration of zinc in DSPs-Zn did not vary substantially between 40 and 80 min, as can be seen in Figure 1C. This indicates that the DSPs’ ability to chelate was unaffected by the reaction’s length. One important factor affecting the zinc concentration in DSPs-Zn is the peptide-zinc mass ratio, as illustrated in Figure 1D. Within the 1:0.5–1:3 range, the zinc concentration in DSPs-Zn rose from 124.39 mg/g to 168.47 mg/g. A decrease in zinc concentration was noted when the mass percentage of peptide to zinc surpassed 1:3. It came to light that the mass of zinc was insufficient to fulfill the chelation reaction’s requirements, and the excess of zinc exceeds the effective binding capacity of Zn^2+^ to the ligand group, resulting in an insignificant chelation reaction and a decrease in the utilization of DSPs [27]. Following a battery of empirical testing, the optimum conditions for the chelation process were determined to be pH 6.0, temperature of 60 degrees Celsius, duration of 60 min, and peptide-zinc mass ratio of 1:3, which resulted in a zinc content of 168.47 mg/g for DSPs-Zn.

#### 3.1.2. Response Surface Optimization

**Figure 2:**
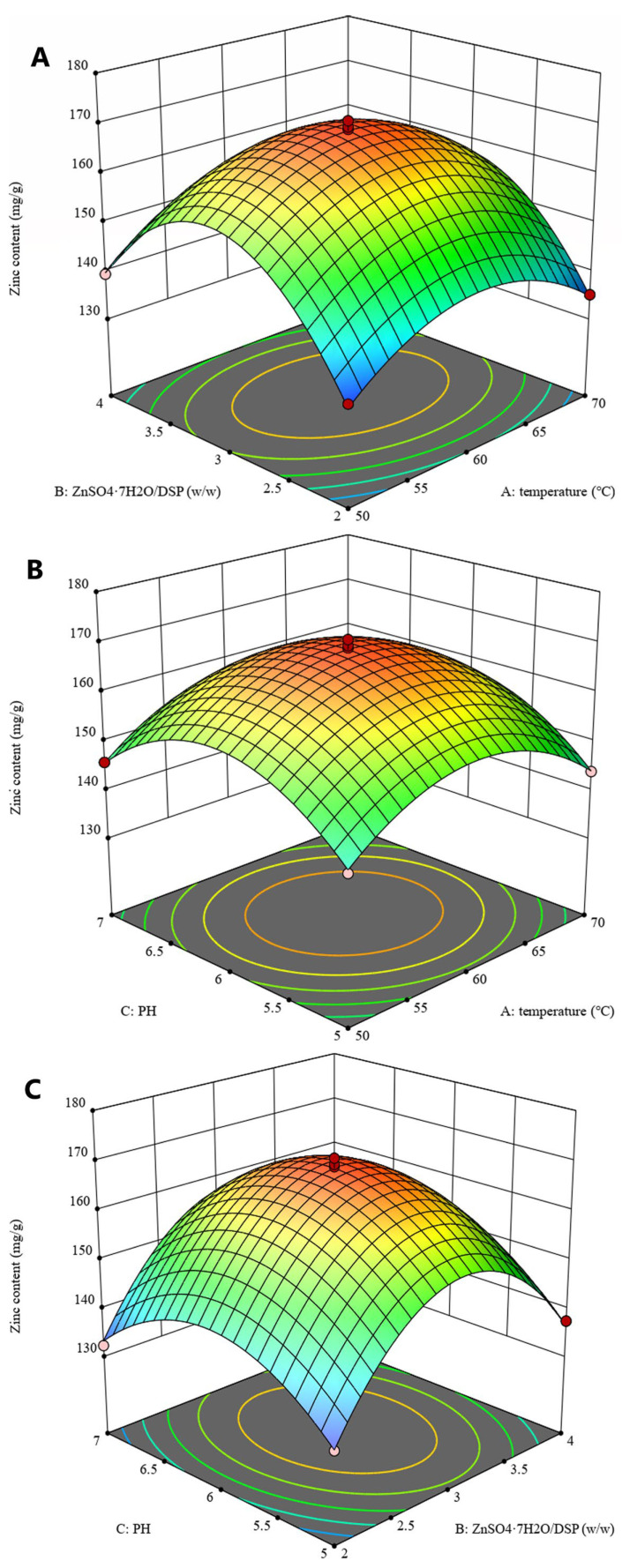
Response surface plots of the effects of three factors on zinc content. (**A**) Interaction of A (pH value) and B (mass ratio of DSP: zinc). (**B**) Interaction of A (pH value) and C (temperature). (**C**) Interaction of B (mass ratio of DSP: zinc) and C (temperature).

The pH, peptide-zinc mass ratio, and temperature were found to significantly influence the zinc content of the DSPs-Zn, and were selected for response surface optimization tests through a one-factor test, and the extreme and centroid values of the factors were determined as indicated in Table 1. A Box-Behnken design (BBD) featuring three factors and three levels was implemented, comprising a total of 17 experimental runs. Table 2 displays the analysis of variance (ANOVA) results. Less than 0.0001 was the *p*-value for the model calculated, and there was no significant correlation between the underfitting (0.7012) relative to the pure error, indicating that the model demonstrated a strong alignment with the experimental data [28]. The coefficient of determination (R^2^ = 0.9954) was found to be highly satisfactory, exhibiting a minimal experimental error and signifying that the model exhibited a strong fit with the response. As demonstrated by the model, the ratio of peptide to zinc (B) exhibited a statistically significant influence on zinc content (*p* < 0.001). Additionally, pH (C) showed a significant influence, with a *p*-value below 0.05. As illustrated in Figure 2, three-dimensional reaction landscape charts demonstrate how variable pairings interact with each of the three components. The following is the expression for the second-degree linear regression equation related to zinc content:

Y= + 168.67 + 0.4675 × A + 2.29 × B + 1.28 × C − 0.55 × AB + 0.225 × AC + 0.6525 × BC − 11.17 × A^2^ − 20.77 × B^2^ − 12.38 × C^2^

The optimum test parameters for the model were pH 6.054, peptide to zinc mass ratio 1:3.071, chelation temperature 60.201 °C, and maximum zinc content of 168.81 mg/g.

The results of the arithmetic example verified the validity of the model. Together with the actual procedure, the expected values were adjusted to account for a pH of 6, a peptide-zinc mass percentage of 1:3, a chelation temperature equal to 60 °C, and a chelation period of 60 min. In these circumstances, at 168.94 mg/g, the actual zinc concentration of the DSPs-Zn was found to be somewhat higher than the expected value. The zinc binding capacity of the oyster peptide synthesized by Peng et al. [29] was 101.08 ± 3.10 mg/g. Hydrolyzed mung bean protein prepared by Fu et al. [30] had a zinc binding potential of 80.82 mg/g. Zinc chelating peptide extracted from Antarctic krill by Sun et al. [31] showed a zinc concentration of 115.70 ± 2.25 mg/g. In addition, the DSPs peptides VPGPMGPSGPR, GDAGPAGPK, GPAGPAGGPR, and GPVGPAG were analyzed in the study reported by Sun et al. [32] with triple repeats of the (Gly-X-Y) primary structure of the collagen peptide chain, which has a high affinity for metal ions. In conclusion, these findings imply that DSPs excellent ability to chelate zinc ions.

### 3.2. Characterization of DSPs and DSPs-Zn

#### 3.2.1. UV-Vis Analysis

**Figure 3:**
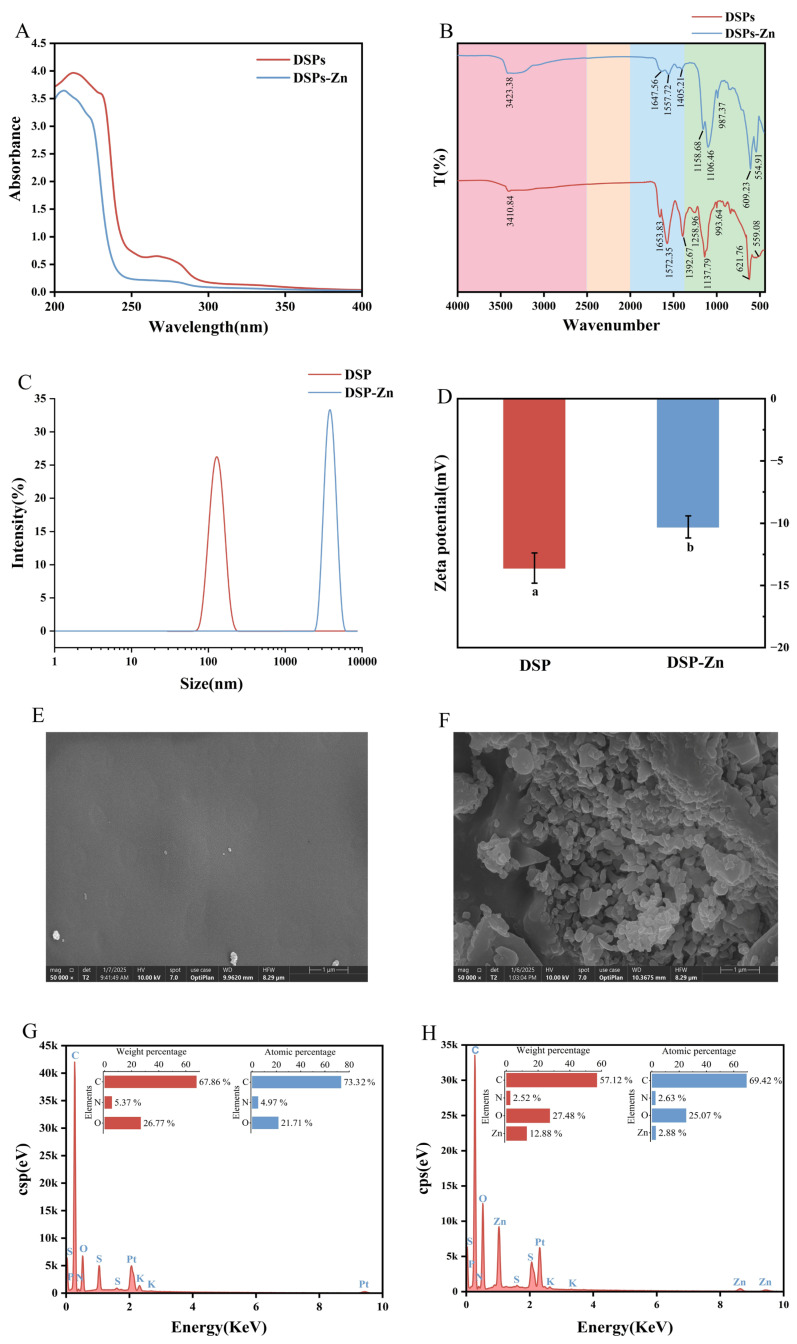
(**A**) UV-vis spectra, (**B**) FTIR spectra, (**C**) particle size distribution, (**D**) zeta potential, (**E**) scanning electron microscopy of DSPs and (**F**) DSPs-Zn; (**G**) surface elemental composition of DSPs and (**H**) DSPs-Zn. Different letters indicate statistically significant differences between groups (*p* < 0.05).

Using the UV-visible spectrum created by the substance’s molecules or ions and the degree of absorption, it has become possible to describe a substance’s structure. When an organic ligand combines with a metal zinc ion, it causes the original absorption peaks to shift, disappear, or appear as new absorption peaks. Differences between DSPs and DSPs-Zn can be analyzed and inferred from the intensity and dislocation changes in the UV absorption spectrum [33]. Amino acid molecules without benzene rings are mainly associated with absorption peaks between 200 and 220 nm, whereas molecules containing aromatic rings are mainly associated with absorption maxima between 250 and 280 nm [34]. For both DSPs and DSPs-Zn, the greatest absorption peaks are found between 200 and 220 nm (Figure 3A). More precisely, DSPs-Zn maximum appears at 206 nm, whilst the DSPs peak is at 212 nm. Measured blueshift of the DSPs complexed with zinc ions is suggested by this change in the absorption peaks. One possible explanation for the finding is that a ligand bond is created when DSPs are chelated with oxygen, nitrogen, and zinc in the peptide structure. This interaction appears to influence the carbonyl group of the peptide bonds (C=O), potentially influencing the n→π * electron transition of the peptide bond [35]. Since zinc ions interact with aromatic amino acids found in DSPs to affect the π→π * electronic transitions of conjugated double bonds, the spectrum of DSPs-Zn between 250 and 280 nm is flatter than that of DSPs [36]. The results presented demonstrate that the sequestration between DSPs and zinc ions involves certain metal binding sites.

#### 3.2.2. FT-IR Analysis

FTIR spectroscopy allows for efficient differentiation between different chemicals based on their differences in spectrum by examining a substance’s composition and determining its functional groups. Upon chelation with zinc ions, the absorption peak shifted from 3410.84 cm^−1^ to 3423.38 cm^−1^ due to the stretching vibration linked to the N-H bond in DSPs (Figure 3B). The occurrence of the N-Zn complex is implied to increase the electron cloud density of the N-H bond [37]. In the double bond stretching vibrational region, which is connected to the amide I band C=O bond stretching, the absorption peaks of DSPs chelated with zinc ions moved from 1653.83 cm^−1^ to 1647.56 cm^−1^; The N-H and C-N stretching vibrations may be the cause of the shift in the absorption peaks of DSPs’ amide bands II and III from 1572.35 cm^−1^ and 1392.67 cm^−1^ to 1557.72 cm^−1^ and 1405.21 cm^−1^ [38]. Within the single bond stretching vibrational region, which spans the spectral range of 1300 cm^−1^ to 900 cm^−1^, the amide bond’s involvement in the chelation reaction is clearly visible. From their initial locations at 1258.96 cm^−1^ and 1137.79 cm^−1^, the DSPs’ absorption peaks showed a shift to new locations at 1158.68 cm^−1^ and 1106.46 cm^−1^. This alteration may be attributed to the C-O stretching and O-H deformation vibrations, as well as the formation of C-O-Zn interactions [39]. Furthermore, because of the N-Zn link’s stretching vibration, the absorption peak at 559.08 cm^−1^ was moved to 554.91 cm^−1^.

#### 3.2.3. Analysis of Particle Size and Zeta Potential

DSPs-Zn had a substantially larger particle size (3016.75 ± 121.65 nm) than DSPs (1655.37 ± 24.77 nm) (Figure 3C), and the difference in size could be caused by intermolecular interactions [40]. Additionally, the two substances were consistently distributed, as indicated by their respective dispersion indices (PDIs) of 0.45 ± 0.03 for DSPs and 0.22 ± 0.01 for DSPs-Zn. Both DSPs and DSPs-Zn showed negative potentials (Figure 3D), and after chelation with zinc ions, the zeta potential increased from −13.60 ± 1.21 mV to −10.30 ± 0.88 mV (*p* < 0.05), a phenomenon that can be attributed to the interaction between negatively charged DSPs and positively charged zinc ions, which resulted in the zeta potential of DSPs-Zn complexes enhancement, which in turn leads to the formation of a novel compound.

#### 3.2.4. Scanning Electron Microscopy and Elemental Composition Analysis

Scanning electron microscopy can characterize the microscopic morphology of substances very intuitively, and the microstructures of DSPs and DSPs-Zn are shown in Figure 3E,F. The surfaces of DSPs are smooth and dense, whereas the surfaces of DSPs-Zn are rough and loose and show irregular shapes. This might be the result of DSPs’ chelation with zinc ions, which alters their initial structure and causes irregular blocks to develop [41]. The elemental compositions of DSPs and DSPs-Zn are shown in Figure 3G,H. The elemental mass percentages of C, N, and O for DSPs were 67.86%, 5.37%, and 26.77%, respectively, and those of C, N, O, and Zn for DSPs-Zn were 57.12%, 2.52%, 27.48%, and 12.88%, respectively. In addition, compared with DSPs, DSPs-Zn exhibited three completely different zinc peaks with significantly enhanced signal intensity and zinc content. The above results indicated that Zn was successfully chelated with DSPs to form new substances after the reaction.

#### 3.2.5. Molecular Weight Distribution

Peptide molecular weight distribution is crucial for chelation. Huang et al. [42] reported that small molecular peptides < 1000 Da have better chelating activity. Table 3 presents the molecular weight distributions for both DSPs and DSPs-Zn. The molecular weight of DSPs < 1000 Da accounted for 60.47 ± 0.05%, indicating that DSPs have strong metal chelating ability. In addition, after chelation with zinc, the percentage of DSPs-Zn chelates with molecular weight < 1000 Da decreased significantly (*p* < 0.05), and the percentage of DSPs-Zn chelates with molecular weight > 1000 Da increased significantly (*p* < 0.05), which explains the reason for the larger particle size distribution.

#### 3.2.6. Amino Acid Composition

The composition of peptide amino acids plays an important role in peptide function. As demonstrated by Table 4, upon zinc chelation, the concentrations of aspartic acid, glutamic acid, lysine, and arginine in DSPs-Zn rose by 2.13%, 0.75%, 0.63%, and 1.06%, respectively, in comparison to DSPs. Aspartic acid and glutamic acid have free carboxyl groups, which is primarily responsible for the synthesis of peptide-zinc chelates following their chelation reaction with zinc ions [43]. Lysine binds to zinc ions because of the presence of the amino acid nitrogen [44]. Chelation of arginine with zinc ions may be related to both thermodynamic stability [45]. According to the outcomes of the testing of amino acid content, arginine, lysine, glutamic acid, and aspartic acid in DSPs are crucial for the zinc chelation process.

### 3.3. Solubility Analysis of Zinc at Different pH Conditions

**Figure 4:**
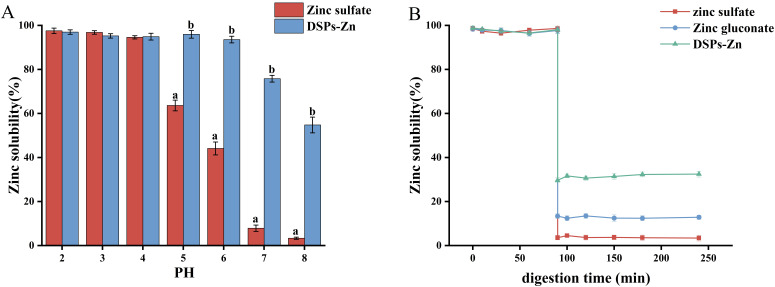
Zinc solubility of DSPs-Zn at different pH (**A**) and simulated gastrointestinal digestion (**B**). Note: DSPs-Zn, deer gluten peptide zinc chelates. Different letters indicate statistically significant differences between groups (*p* < 0.05).

By setting up different pH solutions, the solubility of zinc under different pH conditions can be investigated to verify its stability. Figure 4A illustrates the solubility of zinc from zinc sulfate and DSPs-Zn across a pH range of 2 to 8. Both zinc sulfate and DSPs-Zn had high zinc solubility at pH 2–4 with no significant difference (*p* > 0.05). In the range of 5–8, the solubility of zinc in DSPs-Zn was found to be more soluble in DSPs-Zn than zinc sulfate (*p* < 0.05). Under alkaline conditions, zinc sulfate reacts with OH- in solution to form insoluble zinc salts, which results in a reduction of zinc solubility, whereas DSPs-Zn effectively protects zinc ions from precipitation reaction with OH-, which may be related to its own formed structure [46]. Consequently, the aforementioned findings suggest that DSPs-Zn chelates exhibit favorable solubility and stability in alkaline environments, thereby enhancing the bioavailability of zinc within the human gastrointestinal system.

### 3.4. Simulated Gastrointestinal Digestion Experiment

The stability of DSPs-Zn chelates in the gastrointestinal tract was assessed using zinc sulfate and zinc gluconate as controls. As shown in Figure 4B, the solubility of all three was consistent during the gastric digestion phase from 0 to 90 min. Nonetheless, it appears that solubility in the gut varied dramatically. Specifically, the solubility of zinc sulfate experienced a substantial decline, measuring at 3.56 ± 1.02%, which was markedly lower than zinc gluconate’s (*p* < 0.05), similar to the findings of Li et al. [47]. Throughout the intestinal digestion phase (90–240 min), the solubility of zinc in DSPs-Zn was continuously maintained at elevated levels, indicating a higher level of stability of DSPs-Zn in the setting of gastrointestinal digestion than zinc gluconate and zinc sulfate (*p* < 0.05) [48]. According to the results, zinc ion stability during gastrointestinal digestion is improved when dietary supplement proteins (DSPs) are chelated with zinc ions.

## 4. Conclusions

In the current investigation, deer tendon peptides were utilized to synthesize DSPs-Zn through response surface methodology. The optimal preparation conditions yielded a zinc content of 168.94 mg/g. The successful production of DSPs-Zn was discovered to depend on the existence of amino nitrogen atoms and carboxyl oxygen in the deer tendon peptides. Once the zinc ions have been chelated, significant alterations were observed in the particle size, zeta potential, microstructure, surface elemental composition, molecular weight distribution, and amino acid composition, thereby confirming the chelation of DSPs with zinc ions. Furthermore, DSPs-Zn demonstrated superior zinc solubility than zinc gluconate and zinc sulfate at different pH values and in a laboratory setting that mimics gastrointestinal digestion. This research indicates that DSPs-zinc can be used as a functional food, a novel zinc nutritional supplement with the potential to enhance zinc bioavailability. Notwithstanding the constraints of our investigation, we will further delve into this in subsequent studies to isolate peptide sequences with efficient chelating activity, reveal the structure–activity relationship, and further validate their bioavailability through in vivo and in vitro Caco-2 cell assays.

## Figures and Tables

**Table 1:** Experimental data for zinc content from the Box-Behnken Design.

Coded Levels of Variables				
No.	A: Temperature/°C	B: Mass Ratio of Zinc: Peptide	C: PH	Zinc Content mg/g
1	50	2	6	133.67
2	60	3	6	166.12
3	60	2	5	131.83
4	60	3	6	169.43
5	70	3	7	147.76
6	50	4	6	139.45
7	60	4	7	140.52
8	50	3	7	145.85
9	50	3	5	142.94
10	70	3	5	143.95
11	60	3	6	168.21
12	70	4	6	138.76
13	60	4	5	137.46
14	60	3	6	168.95
15	60	2	7	132.28
16	70	2	6	135.12
17	60	3	6	170.65

**Table 2:** Regression equation variance analysis of zinc content.

Source	Sum of Squares	df	Mean Square	F-Value	*p*-Value	Significant
Modle	3385.60	9	376.18	169.46	<0.0001	**
A	1.75	1	1.75	0.79	0.4043	
B	68.15	1	68.15	30.70	0.0009	**
C	13.08	1	13.08	5.89	0.0456	*
AB	1.21	1	1.21	0.50	0.4845	
AC	0.20	1	0.20	0.09	0.7714	
BC	1.70	1	1.70	0.78	0.4101	
A^2^	525.08	1	525.08	236.54	<0.0001	**
B^2^	1816.35	1	1816.35	818.24	<0.0001	**
C^2^	645.30	1	645.30	290.70	<0.0001	**
Residual	15.54	7	2.22			
Lack of Fit	4.25	3	1.42	0.50	0.7012	not significant
Pure Error	11.29	4	2.82			
Cor Total	3401.14	16	R^2^	0.9954		

* Significant (*p* < 0.05). ** Highly significant (*p* < 0.01).

**Table 3:** Molecular weight distributions of the DSPs and the DSPs-Zn chelate.

Molecular Weight (Da)	DSPs (%)	DSPs-Zn (%)
>10,000	0.05 ± 0.01	0.07 ± 0.01
10,000–5000	7.69 ± 0.06	11.29 ± 0.56 **
5000–3000	8.95 ± 0.07	17.48 ± 0.75 **
3000–1000	22.84 ± 0.16	31.69 ± 0.48 **
1000–500	35.91 ± 0.23	24.45 ± 0.14 **
<500	24.56 ± 0.18	15.02 ± 0.26 **

Note: DSPs, deer sinew peptides; DSPs-Zn, deer sinew peptide zinc chelates. ** Highly significant (*p* < 0.01).

**Table 4:** Amino acid analysis of DSPs and DSPs-Zn.

Amino Acid	Composition (%)	
	DSPs	DSPs-Zn
Asp	6.46 ± 0.06	8.59 ± 0.06 **
Thr	2.14 ± 0.15	1.46 ± 0.14 *
Ser	3.74 ± 0.11	3.58 ± 0.19
Glu	10.71 ± 0.18	11.46 ± 0.13 **
Gly	22.31 ± 0.17	22.28 ± 0.21
Ala	10.34 ± 0.10	10.56 ± 0.12
Val	2.43 ± 0.11	2.08 ± 0.12 *
Met	3.29 ± 0.08	3.52 ± 0.10
Ile	1.27 ± 0.13	0.95 ± 0.15
Leu	2.98 ± 0.10	2.04 ± 0.11 **
Tyr	4.54 ± 0.16	4.65 ± 0.19
Phe	1.84 ± 0.11	1.59 ± 0.10
Lys	3.11 ± 0.12	3.74 ± 0.16 **
His	0.69 ± 0.15	0.47 ± 0.14 **
Arg	8.12 ± 0.14	9.18 ± 0.11 **
Pro	15.28 ± 0.15	14.31 ± 0.22 **
acidic amino acids (Glu and Asp)	17.27 ± 0.24	20.14 ± 0.26 **
alkaline amino acids (Lys, Arg and His)	11.98 ± 0.21	13.39 ± 0.14 **

Note: DSPs, deer sinew peptides; DSPs-Zn, deer sinew peptide zinc chelates. * Significant (*p* < 0.05). ** Highly significant (*p* < 0.01).

## Data Availability

The original contributions presented in the study are included in the article, further inquiries can be directed to the corresponding author.

## Abbreviations

DSPs	deer tendon peptide
DSPs-Zn	deer tendon peptide-zinc chelate
BBD	Box-Behnken Design
UV-vis	ultraviolet-visible absorption spectroscopy
FT-IR	Fourier transform infrared
SEM	scanning electron microscopy
EDS	energy dispersive spectrometer

## References

[1] Huang C., Hightower K.E., Fierke C.A. (2000). Mechanistic Studies of Rat Protein Farnesyltransferase Indicate an Associative Transition State. Biochemistry.

[2] Halsted C.H. (2023). Dietary Supplements and the American Journal of Clinical Nutrition. Am. J. Clin. Nutr..

[3] Costa M.I., Sarmento-Ribeiro A.B., Gonçalves A.C. (2023). Zinc: From Biological Functions to Therapeutic Potential. Int. J. Mol. Sci..

[4] Hussain A., Jiang W., Wang X., Shahid S., Saba N., Ahmad M., Dar A., Masood S.U., Imran M., Mustafa A. (2022). Mechanistic Impact of Zinc Deficiency in Human Development. Front. Nutr..

[5] Gregory P.J., Wahbi A., Adu-Gyamfi J., Heiling M., Gruber R., Joy E.J.M., Broadley M.R. (2017). Approaches to Reduce Zinc and Iron Deficits in Food Systems. Glob. Food Secur..

[6] Maret W., Sandstead H.H. (2006). Zinc Requirements and the Risks and Benefits of Zinc Supplementation. J. Trace Elements Med. Biol..

[7] Sandstead H.H., Smith J.C. (2018). Deliberations and Evaluations of Approaches, Endpoints and Paradigms for Determining Zinc Dietary Recommendations. J. Nutr..

[8] Zhang Z., Zhou F., Liu X., Zhao M. (2018). Particulate Nanocomposite from Oyster (*Crassostrea rivularis*) Hydrolysates Via Zinc Chelation Improves Zinc Solubility and Peptide Activity. Food Chem..

[9] Tanishige H., Yamanaka H., Masuyama R. (2024). Early Regulation of Intestinal Calcium Transport in Response to Luminal Calcium. Clin. Nutr. ESPEN.

[10] Udechukwu M.C., Collins S.A., Udenigwe C.C. (2016). Prospects of Enhancing Dietary Zinc Bioavailability with Food-Derived Zinc-Chelating Peptides. Food Funct..

[11] Guo L., Harnedy P.A., Li B., Hou H., Zhang Z., Zhao X., FitzGerald R.J. (2014). Food Protein-Derived Chelating Peptides: Biofunctional Ingredients for Dietary Mineral Bioavailability Enhancement. Trends Food Sci. Technol..

[12] Miquel E., Farré R. (2007). Effects and Future Trends of Casein Phosphopeptides on Zinc Bioavailability. Trends Food Sci. Technol..

[13] Li J., Gong C., Wang Z., Gao R., Ren J., Zhou X., Wang H., Xu H., Xiao F., Cao Y. (2019). Oyster-Derived Zinc-Binding Peptide Modified by Plastein Reaction via Zinc Chelation Promotes the Intestinal Absorption of Zinc. Mar. Drugs.

[14] Udechukwu M.C., Downey B., Udenigwe C.C. (2017). Influence of Structural and Surface Properties of Whey-Derived Peptides on Zinc-Chelating Capacity, and in Vitro Gastric Stability and Bioaccessibility of the Zinc-Peptide Complexes. Food Chem..

[15] Suo H., Lu L., Zhang L., Zhang X., Li H., Lu Y., Luo X. (2015). Relative Bioavailability of Zinc-Methionine Chelate for Broilers Fed a Conventional Corn–Soybean Meal Diet. Biol. Trace Element Res..

[16] Zhang H., Dong Y., Qi B., Liu L., Zhou G., Bai X., Yang C., Zhao D., Zhao Y. (2014). Preventive Effects of Collagen Peptide from Deer Sinew on Bone Loss in Ovariectomized Rats. Evidence-Based Complement. Altern. Med..

[17] Her C., Thompson A.R., Karim C.B., Thomas D.D. (2018). Probing the Calcium-Dependent Structural States of Calmodulin-RyR using Bifunctional Spin Labels and Deer. Biophys. J..

[18] Wen C., Wang D., Zhang Z., Liu G., Liang L., Liu X., Zhang J., Li Y., Xu X. (2023). Intervention Effects of Deer-Tendon Collagen Hydrolysates on Osteoporosis In Vitro and In Vivo. Molecules.

[19] Cao S., Wang Y., Xing L., Zhang W., Zhou G. (2020). Structure and Physical Properties of Gelatin from Bovine Bone Collagen Influenced by Acid Pretreatment and Pepsin. Food Bioprod. Process..

[20] Xie N., Huang J., Li B., Cheng J., Wang Z., Yin J., Yan X. (2014). Affinity Purification and Characterisation of Zinc Chelating Peptides from Rapeseed Protein Hydrolysates: Possible Contribution of Characteristic Amino Acid Residues. Food Chem..

[21] Xu B., Wang X., Zheng Y., Shi P., Zhang Y., Liu Y., Long N. (2022). Millet Bran Globulin Hydrolysate Derived Tetrapeptide-Ferrous Chelate: Preparation, Structural Characterization, Security Prediction in Silico, and Stability against Different Food Processing Conditions. LWT—Food Sci. Technol..

[22] Na S., Jin Z., Li D., Yin H., Lin S. (2017). An Exploration of the Calcium-Binding Mode of Egg White Peptide, Asp-His-Thr-Lys-Glu, and In Vitro Calcium Absorption Studies of Peptide–Calcium Complex. J. Agric. Food Chem..

[23] Zhao F., Hou W., Guo L., Wang C., Liu Y., Liu X., Min W. (2023). Novel Strategy to the Characterization and Enhance the Glycemic Control Properties of Walnut-Derived Peptides Via Zinc Chelation. Food Chem..

[24] Fang S., Ruan G., Hao J., Regenstein J.M., Wang F. (2020). Characterization and Antioxidant Properties of Manchurian Walnut Meal Hydrolysates After Calcium Chelation. LWT.

[25] Zhai W., Lin D., Mo R., Zou X., Zhang Y., Zhang L., Ge Y. (2023). Process Optimization, Structural Characterization, and Calcium Release Rate Evaluation of Mung Bean Peptides-Calcium Chelate. Foods.

[26] Wu W., He L., Liang Y., Yue L., Peng W., Jin G., Ma M. (2019). Preparation Process Optimization of Pig Bone Collagen Peptide-Calcium Chelate Using Response Surface Methodology and Its Structural Characterization and Stability Analysis. Food Chem..

[27] Huang W., Lan Y., Liao W., Lin L., Liu G., Xu H., Xue J., Guo B., Cao Y., Miao J. (2021). Preparation, Characterization and Biological Activities of Egg White Peptides-Calcium Chelate. LWT.

[28] Cui P., Sun N., Jiang P., Wang D., Lin S. (2017). Optimised Condition for Preparing Sea Cucumber Ovum Hydrolysate–Calcium Complex and Its Structural Analysis. Int. J. Food Sci. Technol..

[29] Peng B., Chen Z., Wang Y. (2023). Preparation and Characterization of an Oyster Peptide–Zinc Complex and Its Antiproliferative Activity on Hepg_2_ Cells. Mar. Drugs.

[30] Fu T., Zhang S., Sheng Y., Feng Y., Jiang Y., Zhang Y., Yu M., Wang C. (2019). Isolation and Characterization of Zinc-Binding Peptides from Mung Bean Protein Hydrolysates. Eur. Food Res. Technol..

[31] Sun R., Liu X., Yu Y., Miao J., Leng K., Gao H. (2020). Preparation Process Optimization, Structural Characterization and In Vitro Digestion Stability Analysis of Antarctic Krill (*Euphausia superba*) Peptides-Zinc Chelate. Food Chem..

[32] Sun L., Liu J., Pei H., Shi M., Chen W., Zong Y., Zhao Y., Li J., Du R., He Z. (2024). Structural Characterisation of Deer Sinew Peptides as Calcium Carriers, Their Promotion of MC3T3-E1 Cell Proliferation and Their Effect on Bone Deposition in Mice. Food Funct..

[33] Zhao L., Huang Q., Huang S., Lin J., Wang S., Huang Y., Hong J., Rao P. (2014). Novel Peptide with a Specific Calcium-Binding Capacity from Whey Protein Hydrolysate and the Possible Chelating Mode. J. Agric. Food Chem..

[34] Qu W., Feng Y., Xiong T., Li Y., Wahia H., Ma H. (2022). Preparation of Corn Ace Inhibitory Peptide-Ferrous Chelate By Dual-Frequency Ultrasound and Its Structure and Stability Analyses. Ultrason. Sonochem..

[35] Wang X., Li M., Li M., Mao X., Zhou J., Ren F. (2011). Preparation and Characteristics of Yak Casein Hydrolysate–Iron Complex. Int. J. Food Sci. Technol..

[36] Xiang H., Huang H., Sun-Waterhouse D., Hu X., Li L., Waterhouse G.I.N., Tang R., Xiong J., Cui C. (2022). Enzymatically Synthesized Γ-[Glu](N≥1)-Gln As Novel Calcium-Binding Peptides to Deliver Calcium with Enhanced Bioavailability. Food Chem..

[37] Wang X., Zhou J., Tong P.S., Mao X.Y. (2011). Zinc-Binding Capacity of Yak Casein Hydrolysate and the Zinc-Releasing Characteristics of Casein Hydrolysate-Zinc Complexes. J. Dairy Sci..

[38] Liao W., Lai T., Chen L., Fu J., Sreenivasan S.T., Yu Z., Ren J. (2016). Synthesis and Characterization of a Walnut Peptides–Zinc Complex and Its Antiproliferative Activity against Human Breast Carcinoma Cells through the Induction of Apoptosis. J. Agric. Food Chem..

[39] Wu J., Cai X., Tang M., Wang S. (2018). Novel Calcium—Chelating Peptides from Octopus Scraps and Their Corresponding Calcium Bioavailability. J. Sci. Food Agric..

[40] Li B., He H., Shi W., Hou T. (2018). Effect of Duck Egg White Peptide-Ferrous Chelate on Iron Bioavailability In Vivo and Structure Characterization. J. Sci. Food Agric..

[41] Wang J., Zhang B., Lu W., Liu J., Zhang W., Wang Y., Ma M., Cao X., Guo Y. (2020). Cell Proliferation Stimulation Ability and Osteogenic Activity of Low Molecular Weight Peptides Derived from Bovine Gelatin Hydrolysates. J. Agric. Food Chem..

[42] Huang G., Ren L., Jiang J. (2010). Purification of a Histidine-Containing Peptide with Calcium Binding Activity from Shrimp Processing Byproducts Hydrolysate. Eur. Food Res. Technol..

[43] Chaud M.V., Izumi C., Nahaal Z., Shuhama T., Bianchi M.d.L.P., de Freitas O. (2002). Iron Derivatives from Casein Hydrolysates as a Potential Source in the Treatment of Iron Deficiency. J. Agric. Food Chem..

[44] Lao L., Jian H., Liao W., Zeng C., Liu G., Cao Y., Miao J. (2023). Casein Calcium-Binding Peptides: Preparation, Characterization, and Promotion of Calcium Uptake in Caco-2 Cell Monolayers. Process. Biochem..

[45] Kállay C., Ősz K., Dávid A., Valastyán Z., Malandrinos G., Hadjiliadis N., Sóvágó I. (2007). Zinc(ii) Binding Ability of Tri-, Tetra- and Penta-Peptides Containing Two or Three Histidyl Residues. Dalton Trans..

[46] Suyin Z., Zheng Y., He S., Su D., Nag A., Zeng Q., Yuan Y. (2020). Novel Zn-Binding Peptide Isolated from Soy Protein Hydrolysates: Purification, Structure, and Digestion. J. Agric. Food Chem..

[47] Li C., Zhou Y., Cao L., Liu Y., Huang Z., Li B., Xu L. (2023). Characterization and Stability of Soybean Meal Peptide-Zinc Chelate Prepared by Solid State Fermentation with *Aspergillus oryzae*. Food Biosci..

[48] Ke X., Hu X., Li L., Yang X., Chen S., Wu Y., Xue C. (2021). A Novel Zinc-Binding Peptide Identified from Tilapia (*Oreochromis Niloticus*) Skin Collagen and Transport Pathway Across Caco-2 Monolayers. Food Biosci..

